# Effect of antidepressant therapy on the severity of COVID 19 symptomatology in the hospitalized patients

**DOI:** 10.1192/j.eurpsy.2023.881

**Published:** 2023-07-19

**Authors:** L. Spirkoska, A. Spasovska Trajkovska, Z. Mitik

**Affiliations:** 1 Psychiatry, GOB 8Septemvri; 2 Psihijatriska Bolnica Skopje; 3 Psychiatry, PZU Zora Mitik, Skopje, North Macedonia

## Abstract

**Introduction:**

Some studies reported that vaccines are extremely good at preventing serious illness but that many countries have had difficulty vaccinating their citizens, and even some vaccinated people may still be at risk for serious COVID-19 symptoms due to underlying medical illness or reduced immunity over time.

**Objectives:**

Some researches reported that drugs known as selective serotonin-reuptake inhibitors (SSRIs) interacts strongly with the sigma-1 receptor, a protein inside cells that helps regulate the body’s inflammatory response. So its believe that this drug most likely is interacting with the sigma-1 receptor to reduce the production of inflammatory molecules in the body, So treatment with this therapy may be highly effective to reducing severe of COVID symptomatology.

**Methods:**

The cross-section study included two groups of patients (N = 30) of different sex ±49,2 age, all were treated in Covid Centre , Skopje. The study was conducted for 6 months all the patients was writing informed consent. The first group consisted of COVID patients who had previously suffered from psychiatric illness and had been treated with antidepressant therapy (SSRi). The second group are the COVID patients who had not been treated with antidepressant before. The severity of COVID 19 symptomatology (based on medical documentation- classification of disease severity) was determined using the Modified Early Warning Score (MEWS scale) . MEWS score 0-2 mild or asymptomatic (stable patients), 3-4 moderately severe (unstable patients), ≥ 5 highly critical(critical patients). The obtained data were processed by descriptive method and Student t-test.

**Results:**

. The results in our study show that the patients who were treated with antidepressants before covid disease showed a lower score on the NEWS scale but there is a not statistically significant results p=0,06 when compare with another examination group

**Conclusions:**

: The results in our study supports considerations about the possible impact of antidepressant therapy on alleviating COVID 19 symptomatology.

**Disclosure of Interest:**

None Declared

